# The role of *PMP22* T118M in Charcot–Marie–Tooth disease remains unsolved

**DOI:** 10.1016/j.jbc.2023.105180

**Published:** 2023-09-11

**Authors:** Christopher J. Record, Matilde Laura, Alexander M. Rossor, Mary M. Reilly

**Affiliations:** Department of Neuromuscular Diseases, UCL Queen Square Institute of Neurology, London, UK

We read with interest the article by Stefanski *et al.* (1) proposing mechanisms by which the T118M variant in *PMP22* predisposes to Charcot–Marie–Tooth (CMT) disease. Previous publications neatly proposed a hereditary neuropathy with liability to pressure palsies–like phenotype in heterozygotes and a more severe neuropathy in homozygotes (2). These, however, did not have the benefit of population databases, showing a heterozygous population prevalence of 1:120 and five homozygotes in GnomADv2 (3). This argues strongly against *PMP22* T188M being a cause of Mendelian disease (4). The authors quote a penetrance of <5% (4.8%), but we would argue that the denominator here is not 1:2500 (the quoted prevalence of CMT) but one quarter of this value (the unsolved 25%) and the theoretical penetrance therefore <1.2%. The experiment showing PMP22 trafficking efficiency was homogenously reduced in T118M, and nondosage dependant, compared with wildtype, was fascinating, but it would be interesting to compare this with the pathogenic L16P and more importantly a known benign variant. Inferring that this phenomenon seen in T118M explains the pathogenicity, without comparison with other variants, is speculative. Curiously, the authors did not look for association between CMT/neuropathy and T118M in the 88,308 patients, which may have been more informative than looking for association with carpal tunnel syndrome. Finally, it is important to clarify that yet no study, including this one, has proven causation or even risk for neuropathy with T118M, as stated in the introduction (5). An exceedingly common variant will have overlap with a common condition like neuropathy, but inferring causation is another matter.

## Conflict of interest

The authors declare that they have no conflicts of interest with the contents of this article.
